# An Opioid Sparing Anesthesia Protocol for Pediatric Open Inguinal Hernia Repair: A Quality Improvement Project

**DOI:** 10.1097/pq9.0000000000000548

**Published:** 2022-03-30

**Authors:** Jennifer L. Chiem, Amber Franz, Nicholas Bishop, David Liston, Daniel K. Low

**Affiliations:** From the Department of Anesthesiology and Pain Medicine. Seattle Children’s Hospital. University of Washington, Seattle, Wash.

## Abstract

Using plan-do-study-act (PDSA) cycles, this quality improvement (QI) project aimed to standardize an anesthetic protocol to optimize multimodal pain management for pediatric open inguinal hernia repair (OIHR).

**Methods:**

PDSA cycle 1: in December 2017, we standardized the intraoperative OIHR anesthesia protocol by replacing transversus abdominis plane (TAP) or ilioinguinal-iliohypogastric (II) blocks and fentanyl with exclusively II blocks and fentanyl. PDSA cycle 2: in January 2019, we used an opioid sparing strategy, replacing II blocks and fentanyl with II blocks and dexmedetomidine. We used statistical process control (SPC) charts to analyze data from the medical record. Outcome measures included the percent of patients requiring rescue morphine in the postanesthesia care unit (PACU), maximum PACU pain score, PACU length of stay (LOS), and anesthesia preparation duration.

**Results:**

The team performed a total of 641 pediatric OIHRs between July 2015 and June 2021. The three groups included 203 patients in our baseline group, 127 patients in the PDSA cycle 1 group, and 311 patients in the PDSA cycle 2 group. Special cause variation (SCV) occurred for the percent of patients requiring rescue morphine, anesthesia preparation duration, and PACU LOS. The percent of patients requiring rescue morphine showed improvement. Anesthesia preparation duration improved compared to baseline. There was no SCV detected in the SPC chart for maximum PACU pain score.

**Conclusion:**

We implemented an opioid sparing anesthetic protocol for pediatric OIHR utilizing II blocks and dexmedetomidine without adversely affecting postoperative pain score or morphine rescue rate over 6 years.

## INTRODUCTION

Inguinal hernia is one of the most common surgical diagnoses in pediatrics. For open inguinal hernia repair (OIHR), ultrasound (U/S)-guided transversus abdominis plane (TAP) and ilioinguinal-iliohypogastric (II) blocks are both utilized for intraoperative and postoperative analgesia.^1,2^ Supplementation with intravenous (IV) opioids both intraoperatively and in the postanesthesia care unit (PACU) is common with both techniques.^1–4^

While the injectate of both TAP and II blocks dissects the plane between the internal oblique and transversus abdominis muscles, the intended target nerves of each are different.^4^ The II block involves blockade of the ilioinguinal and iliohypogastric nerve terminations of the first lumbar nerve root of the lumbar plexus. The TAP block is a fascial plane block that targets a more extensive plexus of nerves and typically requires greater volumes of local anesthetic (LA) to obtain sufficient spread.^5^

Although several studies have compared the efficacy of the two techniques in pediatrics, the results are conflicting.^1–3^ Fredrickson et al.^2^ randomized 41 patients undergoing OIHR to receive either TAP or II blocks. Thirty minutes to 2 hours postoperatively, pain was more frequent and ibuprofen use was higher in the TAP group; morphine consumption was similar between groups. Conversely, Sahin et al. randomized 90 children into three groups: TAP, II, and caudal.^3^ The total amount of perioperative analgesic consumption and pain scores at 1, 4, and 8 hours after surgery were significantly higher in the II group than the other two. The results of randomized controlled trials (RCT) in adults comparing these two blocks for OIHR are similarly mixed.^4,6^

At Seattle Children’s Bellevue Clinic and Surgery Center (BCSC), approximately 100 OIHRs are performed annually. Before 2017, BCSC anesthesiologists administered either a TAP or II block, based on preference, for OIHR, with intraoperative IV fentanyl supplementation. In December 2017, speculation on the utility of TAP blocks for OIHR prompted BCSC anesthesiologists to standardize their practice to perform II blocks exclusively for OIHR.

Concurrently, a local and national shortage of IV opioid medications in 2017–2018 caused BCSC providers to explore opioid sparing pain management strategies to conserve the opioid supply.^7,8^ One methodology utilized dexmedetomidine, a highly selective alpha-2 agonist with both sedating and analgesic properties that binds to receptors in the spinal cord and locus coeruleus.^9^ In the current pediatric literature, dexmedetomidine is used for preoperative anxiety,^10^ procedural sedation,^11,12^ emergence delirium,^13–15^ and intensive care unit sedation.^9^ There are a few reports of intraoperative use for pain control.^16–18^ A quality improvement (QI) project centered on tonsillectomy and adenotonsillectomy surgeries at BCSC showed that replacing intraoperative morphine and acetaminophen with dexmedetomidine and ketorolac resulted in similar pain scores and PACU length of stay (LOS). At the same time, nausea and vomiting rescue rates improved.^18^ RCTs on dexmedetomidine use, specifically in pediatric OIHR, are limited to its use as an adjuvant for regional techniques.^19^

Based upon the BCSC T&A QI project findings, the BCSC facility expanded the use of dexmedetomidine in its protocols, minimizing intraoperative opioids for multiple pediatric ambulatory surgeries in a phased manner to track effectiveness.^18,20^ In January 2019, the team replaced fentanyl with dexmedetomidine in the OIHR protocol.

We aimed to standardize an anesthetic protocol to optimize pain management using a multimodal approach for pediatric OIHR over 6 years. We selected maximum PACU pain score and the percent of patients requiring postoperative rescue morphine as primary outcome measures and PACU LOS and anesthesia preparation duration as balancing measures.

## METHODS

BCSC is an ambulatory outpatient facility where clinicians incorporate evidence-based standardized anesthesia protocols into their practice to optimize delivery of patient care. Anesthesiologists and certified registered nurse anesthetists (CRNAs) can track the adoption and effectiveness of protocol changes due to the healthy patient population, low-acuity, high-volume caseload, and the informatics infrastructure built to capture electronic medical record (EMR) data, which began in 2015.

In this QI project, we included American Society of Anesthesiologists class 1–2 patients 3–18 years of age undergoing OIHR at BCSC from July 1, 2015, through June 30, 2021. We chose this time frame to allow providers time to adhere to multiple protocol changes at the BCSC facility.

Before December 1, 2017, both TAP and II blocks were performed in children aged 3-18 years, depending on the anesthesiologist’s preference. The techniques for performing TAP and II blocks at BCSC mirror those described by the New York School of Regional Anesthesia.^5^ All regional blocks are performed immediately following induction.

### PDSA Cycle 1

The BCSC anesthesia team standardized their practice on December 1, 2017, to perform II blocks exclusively for patients 3–18 years of age undergoing OIHR. Adjunctive intraoperative analgesics included in the protocol during this time period included fentanyl (0.5–1 µg/kg) and ketorolac (0.5 mg/kg).

### PDSA Cycle 2

This cycle began January 1, 2019, in which dexmedetomidine (0.5–1 µg/kg) replaced intraoperative fentanyl (0.5–1 µg/kg) in the OIHR protocol.

Percent of patients requiring postoperative rescue morphine was one of the primary outcome measures used to assess protocol effectiveness. PACU nurses often administer oral acetaminophen for mild pain (score 1–3). Morphine is the first-line rescue analgesic for moderate (score 4–6) to severe (score 7–10) pain in our PACU.

PACU maximum pain score was also a primary outcome measure. PACU nurses record pain scores using assessment tools at their discretion. For patients ≤3 years old, the nurses typically use the Faces, Legs, Activity, Cry, Consolability scale (FLACC; validity *r = 0*.41–0.8, reliability 69%–91%, kappa = 0.52–0.82).^21^ For patients 3–6 years old, the nurses favor the Faces Pain Scale-Revised (FPS-R; validity = 0.84–0.99, interrater correlations = 0.84–0.99).^22^ The nurses commonly use the numerical 0–10 visual analog scale (validity = 0.61–0.90, reliability 0.41–0.58, interrater correlation 0.28–0.72) for patients 7 years of age and older.^22^ Statistical scores presented above are from references; we do not have interrater reliability scores for staff. We converted each pain assessment tool into an 11-point (0–10) score for the analyses.

We examined PACU LOS and anesthesia preparation duration as balancing measures because the literature suggests that II blocks are more technically challenging and more time consuming than TAP blocks.^2^ Dexmedetomidine can also delay arousal and increase time to discharge,^15^ which is not preferable in a high turnover ambulatory surgical center. Anesthesia preparation start time begins with induction and ends after the anesthesia provider completes the peripheral nerve block. PACU LOS begins with the handoff from an anesthesia provider postoperatively and ends with patient discharge from PACU to home. Patients and families were discharged directly from PACU to home once they returned to their preoperative baseline or met discharge criteria based upon the Aldrete scoring system.^23^

AdaptX OR Advisor (AdaptX, Seattle) software system extracts continuously updated, aggregated, and de-identified health information from the hospital’s electronic medical record. It presents the data as statistical process control (SPC) charts.^18^

This QI project used P-charts to display the percent of patients requiring postoperative rescue morphine and X-bar charts to display maximum pain score, PACU LOS, and anesthesia preparation duration.^24,25^ Every X-bar chart has a paired S-chart. Control limits were set at three-sigma above and below the mean. Standard SPC chart rules were used to detect common cause and special cause variation (SCV).^24^

We submitted this QI project to the Seattle Children’s Institutional Review Board. The board considered this QI work and not human subjects research, so there was no further review.

## RESULTS

**Fig. 1.**
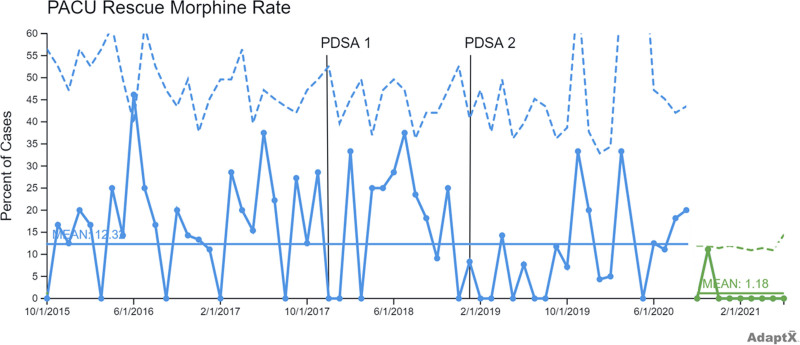
P chart, postoperative morphine rescue rate in PACU. I/D, ilioinguinal-iliohypogastric block/dexmedetomidine; I/F, ilioinguinal-iliohypogastric block/fentanyl; T/F, transversus abdominal plane block/fentanyl.

**Fig. 2.**
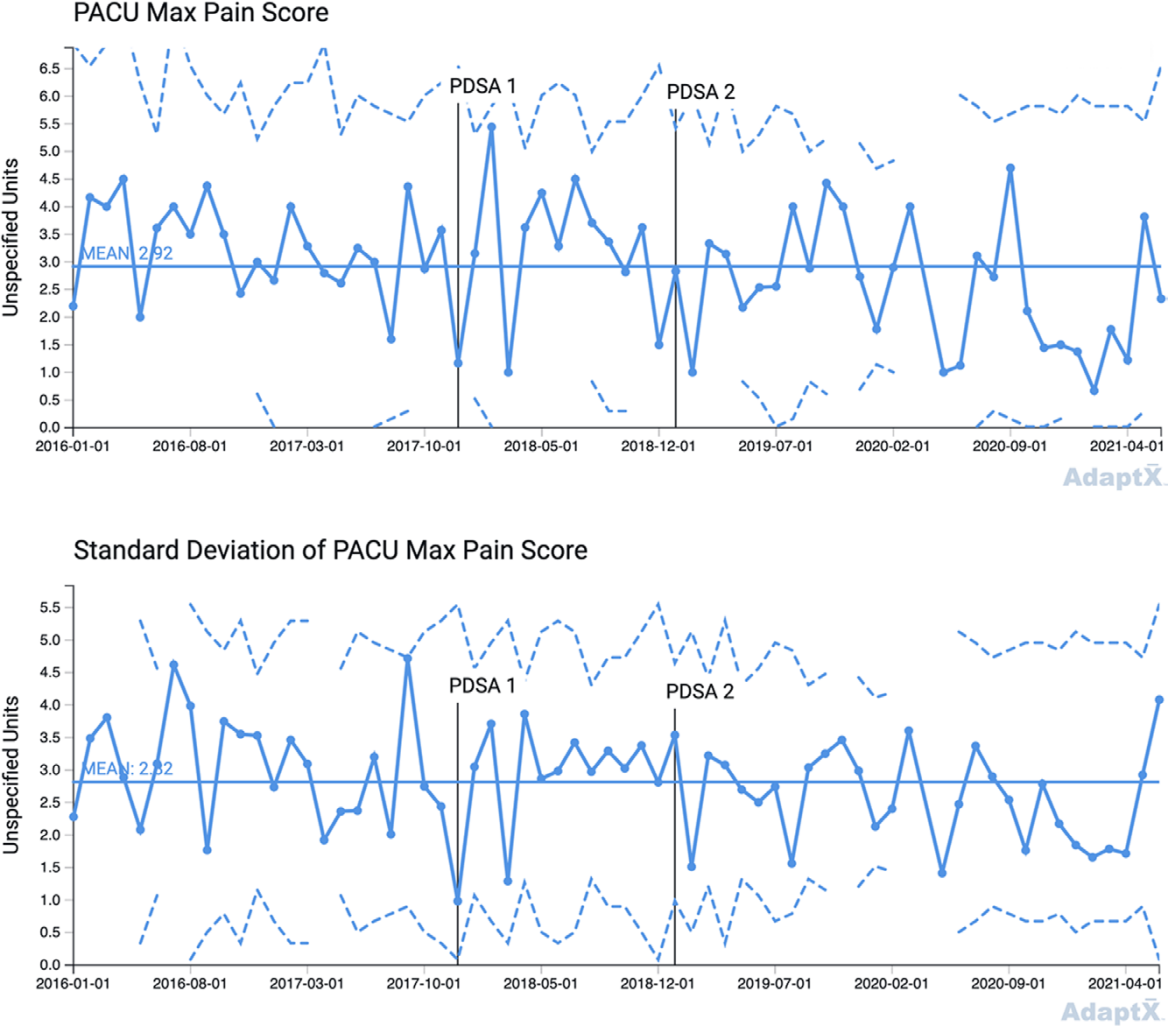
X-bar chart, mean maximum pain score in PACU. I/D, ilioinguinal-iliohypogastric block/dexmedetomidine; I/F, ilioinguinal-iliohypogastric block/fentanyl; T/F, transversus abdominal plane block/fentanyl.

**Fig. 3.**
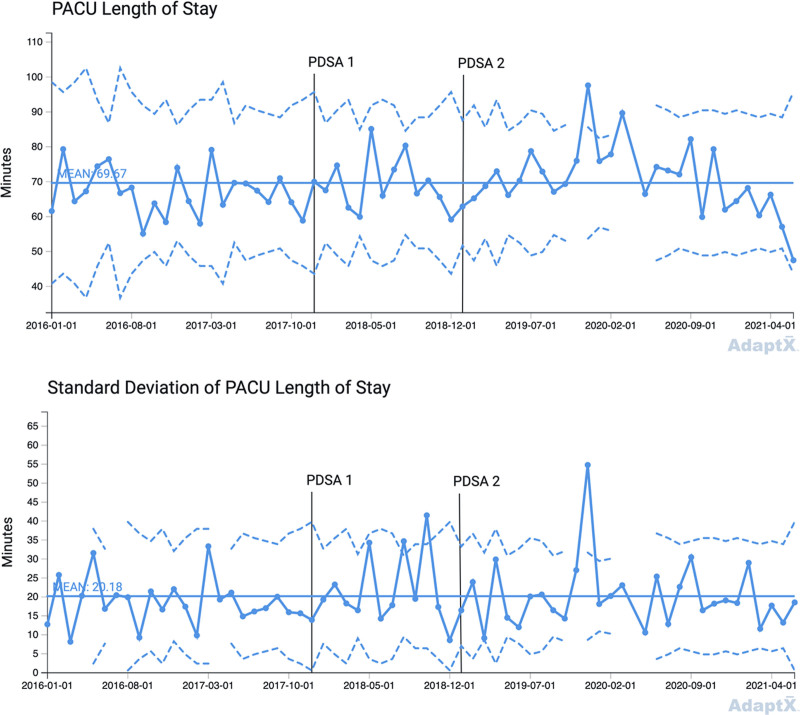
X-bar chart, mean PACU length of stay. I/D, ilioinguinal-iliohypogastric block/dexmedetomidine; I/F, ilioinguinal-iliohypogastric block/fentanyl; T/F, transversus abdominal plane block/fentanyl.

**Fig. 4.**
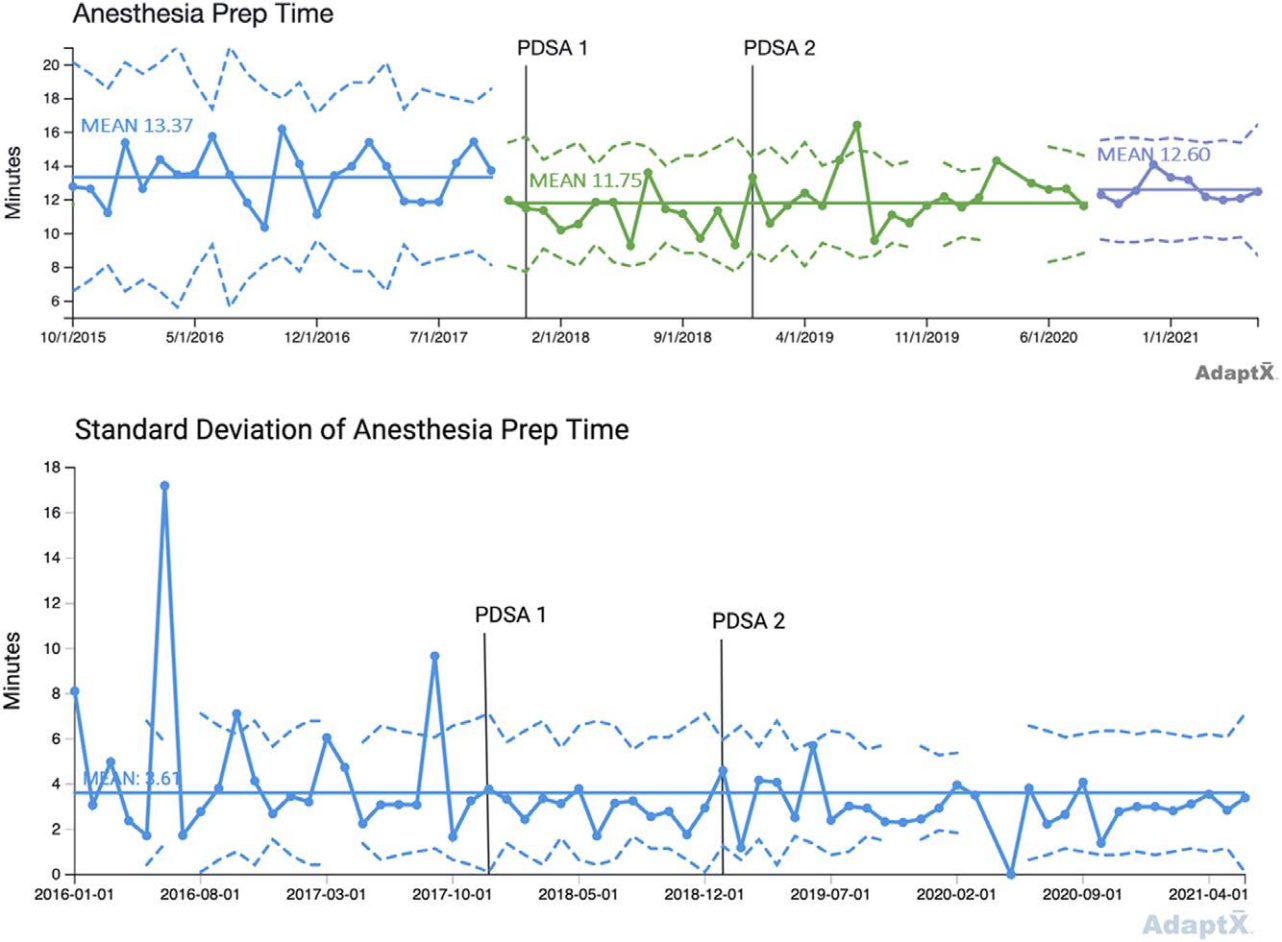
X-bar chart, mean anesthesia preparation duration. I/D, ilioinguinal-iliohypogastric block/dexmedetomidine; I/F, ilioinguinal-iliohypogastric block/fentanyl; T/F, transversus abdominal plane block/fentanyl.

There were 203 patients in the baseline group, 127 in the PDSA cycle 1 protocol group, and 311 in the PDSA cycle 2 protocol group. Before December 1, 2017, TAP blocks were performed for 38% of OIHR cases (78/203), whereas II blocks were performed for 62% of OIHR cases (125/203) (baseline group). From December 1, 2017, to December 31, 2018 (PDSA 1 group), 4% of OIHR cases received a TAP block (5/127) and 96% of OIHR cases received an II block (122/127), whereas 69% of OIHR cases received intraoperative fentanyl (88/127). From January 1, 2019, to June 30, 2021 (PDSA 2 group), 96% of OIHR cases received intraoperative dexmedetomidine (298/311), whereas only 5% of OIHR cases received intraoperative fentanyl (14/311). Table 1 lists demographic data during the PDSA cycles. Table 2 shows changes over time in the anesthesia OIHR protocol. Figures 1–4 display SPC results. In the X-bar charts, months in which three or less procedures were performed do not display control limits as standard deviations could not be calculated. Some control charts have discontinuous control limits because there were months with low case numbers, making it inappropriate to calculate three-sigma limits. For example, during the COVID-19 pandemic, BCSC was closed temporarily in the spring of 2020 per CDC guidelines for elective surgery.

**Table 1.** Patient Demographics

	Baseline (N = 203)	PDSA 1 (N = 127)	PDSA 2 (N = 311)
Sex (N/%)			
Male	126 (62.1%)	71 (55.9%)	214 (68.8%)
Female	77 (37.9%)	56 (44.1%)	97 (31.2%)
Age in Years (mean/range)	6.8 (3–18)	6.6 (3–18)	5.6 (3–17)
BMI in kg/m^2^ (mean/range)	16.5 (12.6–28.5)	16.3 (12.7–27.4)	16.7 (12.5–31.2)
ASA score (N/%)			
1	147 (72.4%)	93 (73.2%)	203 (65.3%)
2	56 (27.6%)	34 (26.8%)	108 (34.7%)
Race (N/%)			
White or Caucasian	112 (55.2%)	70 (55.1%)	164 (52.7%)
Black/African American	7 (3.4%)	2 (1.6%)	4 (1.3%)
Asian	26 (12.8%)	15 (11.8%)	41 (13.2%)
Hispanic	21 (10.3%)	10 (7.9%)	40 (12.9%)
Other	27 (13.3%)	15 (11.8%)	45 (14.5%)
Patient refused	10 (5.0%)	15 (11.8%)	17 (5.4%)

ASA, American Society of Anesthesiologists classification; BMI, body mass index.

**Table 2.** Anesthesia Protocols

Baseline, N = 203	PDSA 1, N = 127	PDSA 2, N = 311
July 1, 2015–November 30, 2017	December 1, 2017–December 31, 2018	January 1, 2019–June 30, 2021
Induction—Sevoflurane 8%/oxygen/nitrous oxide Propofol 1–2 mg/kg IV for LMA placement Maintenance—Sevoflurane 0.8–1.3 MAC/<30% oxygen/air Dexamethasone 0.15 mg/kg IV (max 8 mg) Ondansetron 0.15 mg/kg IV (max 4 mg) Antibiotics, if necessary Ketorolac 0.5 mg/kg IV (max 30 mg) once surgery complete		
TAP block vs II block, 0.5% ropivacaine, 0.2–0.3 ml/kg immediately following induction	II block, 0.5% ropivacaine, 0.1–0.2 ml/kg immediately following induction	II block, 0.5% ropivacaine, 0.1–0.2 ml/kg immediately following induction
Fentanyl 1–2 µg/kg IV PRN	Fentanyl 1–2 µg/kg IV PRN	Dexmedetomidine 0.5 µg/kg IV bolus at induction, additional 0.5 µg/kg PRN

ETT, endotracheal tube; II, ilioinguinal-iliohypogastric; IV, intravenous; LMA, laryngeal mask airway; max, maximum; MAC, minimum alveolar concentration; PRN, pro re nata (as needed); TAP, transversus abdominis plane.

Figure 1 P-chart, displays the percent of patients requiring postoperative rescue morphine. The centerline mean indicates that 12.32% of patients required morphine in the PACU between July 2015 and June 2021. PDSA interventions are annotated on the charts. There is a single point above the upper control limit (UCL) in the baseline group, though the rest of this time period is stable. There is a run of nine points below the centerline mean after PDSA cycle 2, denoted in green, indicating special cause variation and decreased morphine rescue rate. The centerline mean for the run of nine points was 1.18%.

Figure 2 X-bar chart, displays the maximum PACU pain scores. The centerline mean is 2.92, and the measure is stable throughout both PDSA cycles (no SCV signals detected). The accompanying S-chart shows a SD mean of 2.82.

Figure 3 illustrates the X-bar chart for PACU LOS. The X-bar centerline mean is 69.67 minutes and the chart demonstrates process stability except in PDSA group 2—a breach in the UCL in December 2019. The accompanying S-chart shows a SD mean of 20.18 minutes.

Figure 4 depicts the X-bar chart for anesthesia preparation duration. The centerline mean at baseline is 13.37 minutes and the data in this time period are stable. Beginning in September 2017, there is a decreasing trend in anesthesia preparation time, with a shift down from November 2017 to June 2018. This results in a new centerline at 11.75 minutes. After implementation of PDSA cycle 2, there is one breach above the UCL in July 2019. Then, in September 2020, there is an upward shift with a new centerline mean of 12.60 minutes. The accompanying S-chart shows a SD mean of 3.61 minutes over the entire period.

## DISCUSSION

This project consisted of baseline data and the implementation of two anesthesia protocols via two PDSA cycles, assessing the effectiveness of multimodal OIHR care over 6 years. There was a reduction in the percent of patients requiring rescue morphine in PACU after implementation of PDSA cycle 2, with no notable changes to PACU maximum pain score, indicating improvement with our opioid sparing II block and dexmedetomidine protocol.

PACU LOS remained stable with the exception of two signals after implementation of PDSA cycle 2. The breach in December 2019 was due to extended PACU stays for several patients. One patient experienced postoperative nausea and vomiting, leading to an extended PACU stay. Three other patients had PACU stays >120 minutes, though there wasn’t any clear documentation regarding why. The May–June 2021 signal may indicate an improvement in our PACU LOS for OIHR patients, though more data need to be collected to determine if this trend continues.

Anesthesia preparation duration showed several signals. The decreasing trend noted in September 2017 and shift downward starting in November 2017 may be related to increasing comfort and efficiency with II blocks, which were becoming more commonplace during this time, even though the official change from TAP to II blocks did not occur until December 2017. This improvement with the adoption of II blocks lasted over two and a half years during PDSA cycles 1 and 2, but was not sustained. Starting in September 2020, there was a shift upward from the prior mean—this increase is likely due to hiring of several new nurse anesthetists at this time. However, even with this minor shift up (mean 12.60 minutes), the anesthesia prep time remained lower than the baseline group (mean 13.37 minutes), suggesting no real change. Of note, the breach of the UCL in July 2019 was due to two cases where “Anesthesia Start Time” was charted erroneously.

We were able to minimize intraoperative opioids in PDSA cycle 2 by utilizing II blocks and dexmedetomidine in our protocol, while maintaining low rates of moderate to severe pain in our OIHR population. Dexmedetomidine has been well established as an analgesic adjunct and can reduce the need for opioids.^16–18^ This study demonstrated similar results within a pediatric OIHR patient population. A study of 60 children undergoing OIHR randomized to receive II block ± adjunctive dexmedetomidine found prolonged duration of analgesia, lower pain scores, and less rescue analgesic requirements during the first 24 hours postoperatively in the group receiving dexmedetomidine.^19^ While this study supports the use of II block + dexmedetomidine for OIHR versus II block alone, it does not include an assessment of II block + fentanyl, as was done in our QI study.

Prior pediatric literature suggests that II blocks are more technically challenging and time consuming than TAP blocks.^2^ At first glance, our results seem to differ, as anesthesia preparation duration time actually improved for several years when II blocks became the standard for OIHR. However, before PDSA cycle 1, most of our OIHR patients were already receiving II blocks (62%)—our reduced preparation time may thus be due to improved skill and efficiency as providers performed this block more frequently.

Dexmedetomidine has the potential to prolong emergence and PACU LOS in the perioperative period.^15^ However, stability in our PACU LOS X-bar chart indicated no deterioration in this measure once dexmedetomidine was instituted in the protocol. This may be because dexmedetomidine is common in several other protocols at BCSC and our practitioners and nurses are already comfortable with the use and pharmacokinetics of this medication.

This study has several limitations. First, despite the implementation of prescribed OIHR protocols in PDSA cycles 1 and 2, we did not have complete uptake of each of the relevant protocol changes by our team (process measure), possibly leading to less clear results. Second, though there is correlation between pain scores from different pain assessment tools, we converted scores from three different pain assessment tools into an 11-point (0–10) scale in a nonvalidated manner.^26^ This is not ideal since pain may be assessed differently depending on who evaluates the patient’s pain level.^27^ However, when separating patients by age (3, 3–6, and 7–18 years) and thus grouping patients with similar pain assessment tool use, we found no SCV within any of the age groups for maximum PACU pain score over the 6-year project. We believe this is consistent with our combined pain scale results. Third, we did not examine hemodynamic data, such as hypotension and bradycardia (common side effects of dexmedetomidine) to determine potential limitations with its use in the pediatric population. However, ephedrine, glycopyrrolate, and atropine administration can be used as proxies for treatment of hypotension and bradycardia. In our baseline group, PDSA 1 group, and PDSA 2 group, 2%, 0%, and 5% received ephedrine, whereas 1%, 2%, and 1% received glycopyrrolate, respectively. One patient received atropine in the PDSA 1 group. Last, a limitation of many QI projects is that, while validated at their home institutions, they are not necessarily generalizable. Our team has extensive experience with anesthesia protocol implementation, dexmedetomidine administration, opioid minimization, and regional blocks, and access to a software program that extracts and displays continuously updated data from the hospital’s EMR in the form of SPC charts—these factors may not be the norm at other institutions. Regardless, this study serves as an example of how readily accessible EMR data can support QI work, encourage adoption of standardized clinical protocols, and reduce variation in practice to improve the quality of clinical care.

## CONCLUSION

By leveraging PDSA cycles, real-time EMR data, and SPC charts, we optimized an anesthesia protocol for OIHR surgery utilizing II blocks and dexmedetomidine that minimized the need for intraoperative opioids while decreasing the percent of patients requiring postoperative rescue morphine. Anesthesia preparation duration improved compared to baseline data. Further research, such as a multicenter, randomized controlled trial is needed to determine if our findings are relevant for other institutions.

## DISCLOSURE

Dr. Daniel Low is the Chief Medical Officer and founder of AdaptX, and Dr. David Liston is a shareholder in AdaptX. The other authors have no financial interest to declare in relation to the content of this article.

## References

[1] Fredrickson M Seal P Houghton J. Early experience with the transversus abdominis plane block in children. Paediatr Anaesth. 2008;18:891–892. 18768050 10.1111/j.1460-9592.2008.02591.x

[2] Fredrickson M Paine C Hamill J. Improved analgesia with ilioinguinal block compared to the transversus abdominis plane block after pediatric inguinal surgery: a prospective randomized trial. Pediatr Anesth. 2010;20:1022 1027. 10.1111/j.1460-9592.2010.03432.x 20964768

[3] Sahin L Soydinc MH Sen E. Comparison of 3 different regional block techniques in pediatric patients. A prospective randomized single-blinded study. Saudi Med J. 2017;38:952–959. 28889155 10.15537/smj.2017.9.20505 PMC5654031

[4] Stav A Reytman L Stav MY. Transversus abdominis plane versus ilioinguinal and iliohypogastric nerve blocks for analgesia following open inguinal herniorrhaphy. Rambam Maimonides Med J. 2016;7:e0021. 10.5041/RMMJ.10248 PMC5001793 27487311

[5] NYSORA. Ultrasound-Guided Transversus Abdominis Plane and Quadratus Lumborum Blocks. Available at https://www.nysora.com/regional-anesthesia-for-specific-surgical-procedures/abdomen/ultrasound-guided-transversus-abdominis-plane-quadratus-lumborum-blocks/. Accessed May 27, 2019.

[6] Aveline C Le Hetet H Le Roux A. Comparison between ultrasound-guided transversus abdominis plane and conventional ilioinguinal/iliohypogastric nerve blocks for day-case open inguinal hernia repair. Br J Anaesth. 2011;106:380–386. 21177284 10.1093/bja/aeq363

[7] U.S. Food and Drug Administration. Statement from Douglas Throckmorton, M.D., Deputy Center Director for Regulatory Programs in FDA’s Center for Drug Evaluation and Research, on the Agency’s Response to Ongoing Drug Shortages for Critical Products. Available at https://www.fda.gov/NewsEvents/Newsroom/PressAnnouncements/ucm611215.htm. 2018. Accessed October 6, 2019.

[8] Hollingsworth H Herndon C. The parenteral opioid shortage: causes and solutions. J Opioid Manag. 2018;14:81–82. 29733093 10.5055/jom.2018.0434

[9] Kaur M Singh PM. Current role of dexmedetomidine in clinical anesthesia and intensive care. Anesth Essays Res. 2011;5:128–133. 25885374 10.4103/0259-1162.94750 PMC4173414

[10] Sajid B Mohamed T Jumaila M. A comparison of oral dexmedetomidine and oral midazolam as premedicants in children. J Anaesthesiol Clin Pharmacol. 2019;35:36–40. 31057237 10.4103/joacp.JOACP_20_18 PMC6495609

[11] Schacherer NM Armstrong T Perkins AM. Propofol versus Dexmedetomidine for procedural sedation in a pediatric population. South Med J. 2019;112:277–282. 31050796 10.14423/SMJ.0000000000000973

[12] Neville DN Hayes KR Ivan Y. Double-blind randomized controlled trial of intranasal Dexmedetomidine versus intranasal Midazolam as anxiolysis prior to pediatric laceration repair in the emergency department. Acad Emerg Med. 2016;23:910–917. 27129606 10.1111/acem.12998

[13] Begum U Singh PR Naithani B. Dexmedetomidine as Bolus or low-dose infusion for the prevention of emergence agitation with sevoflurane anesthesia in pediatric patients. Anesth Essays Res. 2019;13:57–62. 31031481 10.4103/aer.AER_177_18 PMC6444969

[14] Shi M Miao S Gu T. Dexmedetomidine for the prevention of emergence delirium and postoperative behavioral changes in pediatric patients with sevoflurane anesthesia: a double-blind, randomized trial. Drug Des Devel Ther. 2019;13:897–905. 10.2147/DDDT.S196075 PMC6421876 30936683

[15] Zhu M Wang H Zhu A. Meta-analysis of dexmedetomidine on emergence agitation and recovery profiles in children after sevoflurane anesthesia: different administration and different dosage. PLoS ONE. 2015;10:1 22. 10.1371/journal.pone.0123728 PMC4395116 25874562

[16] Olutoye OA Glover CD Diefenderfer JW. The effect of intraoperative dexmedetomidine on postoperative analgesia and sedation in pediatric patients undergoing tonsillectomy and adenoidectomy. Anesth Analg. 2010;111:490–495. 20610555 10.1213/ANE.0b013e3181e33429

[17] Blaudszun G Lysakowski C Elia N. Effect of perioperative systemic α2 agonists on postoperative morphine consumption and pain intensity: systematic review and meta-analysis of randomized controlled trials. Anesthesiology. 2012;116:1312–1322. 22546966 10.1097/ALN.0b013e31825681cb

[18] Franz AM Dahl JP Huang H. The development of an opioid sparing anesthesia protocol for pediatric ambulatory tonsillectomy and adenotonsillectomy surgery-A quality improvement project. Paediatr Anaesth. 2019;29:682–689. 31077491 10.1111/pan.13662

[19] Karan D Swaro S Mahapatra PR. Effect of Dexmedetomidine as an adjuvant to Ropivacaine in ilioinguinal-iliohypogastric nerve blocks for inguinal hernia repair in pediatric patients: a randomized, double-blind, control trial. Anesth Essays Res. 2018;12:924–929. 30662132 10.4103/aer.AER_169_18 PMC6319075

[20] Franz AM Martin LD Liston DE. In pursuit of an opioid-free pediatric ambulatory surgery center: a quality improvement initiative. Anesth Analg. 2021;132:788 797. 32282383 10.1213/ANE.0000000000004774

[21] Merkel SI Voepel-Lewis T Shayevitz JR. The FLACC: a behavioral scale for scoring postoperative pain in young children. Pediatr Nurs. 1997;23:293–297. 9220806

[22] Cohen LL Lemanek K Blount RL. Evidence-based assessment of pediatric pain. J Pediatr Psychol. 2008;33:939–955. 18024983 10.1093/jpepsy/jsm103 PMC2639489

[23] Aldrete JA. The post-anesthesia recovery score revisited. J Clin Anesth. 1995;7:89–91. 7772368 10.1016/0952-8180(94)00001-k

[24] Vetter TR Morrice D. Statistical process control: no hits, no runs, no errors? Anesth Analg. 2019;128:374–382. 30531221 10.1213/ANE.0000000000003977

[25] Provost L Murray S. The Health Care Data Guide: Learning from Data for Improvement. Jossey-Bass; 2011.

[26] Crellin D Harrison D Santamaria N. Comparison of the psychometric properties of the FLACC scale, the MBPS and the observer applied visual analogue scale used to assess procedural pain. J Pain Res. 2021;14:881–892. 33833566 10.2147/JPR.S267839 PMC8020135

[27] Manne SL Jacobsen PB Redd WH. Assessment of acute pediatric pain: do child self-report, parent ratings, and nurse ratings measure the same phenomenon? Pain. 1992;48:45–52. 1738574 10.1016/0304-3959(92)90130-4

